# The role of oxidative stress and antioxidant approaches in preeclampsia: A narrative review

**DOI:** 10.18332/ejm/216377

**Published:** 2026-01-29

**Authors:** José Juan Quilantán-Cabrera, Luis Fernando López-Ávalos, Selene Guadalupe Huerta-Olvera, Juan Ramón Gómez-Sandoval, José Javier Morales-Núñez, Omar Graciano-Machuca, Sonia Sifuentes-Franco

**Affiliations:** 1 División de Ginecología y Obstetricia, Nuevo Hospital Civil de Guadalajara Dr. Juan I. Menchaca, Guadalajara, México; 2 Departamento de Clínicas de la Reproducción Humana, crecimiento y desarrollo infantil, Centro Universitario de Ciencias de la Salud, Universidad de Guadalajara, Guadalajara, Mexico; 3 Departamento de Ciencias Médicas y de la Vida, Centro Universitario de la Ciénega, Universidad de Guadalajara, Ocotlán, México; 4 Especialidad en Periodoncia, Departamento de Clínicas Odontológicas Integrales, Instituto de Investigación en Odontología, Centro Universitario de Ciencias de la Salud, Universidad de Guadalajara, Guadalajara, México; 5 Instituto de Investigación en Ciencias Biomédicas, Departamento de Biología Molecular y Genómica, Centro Universitario de Ciencias de la Salud, Universidad de Guadalajara, Guadalajara, Mexico; 6 Laboratorio de Ciencias Clínicas, Departamento de Ciencias de la Salud. Centro Universitario de Los Valles, Universidad de Guadalajara, Ameca, México

**Keywords:** preeclampsia, oxidative stress, antioxidants, endothelial dysfunction, inflammatory cytokines

## Abstract

Preeclampsia (PE) is a potentially life-threatening pregnancy complication characterized by new-onset hypertension and proteinuria. Although multiple risk factors have been associated with its development, the underlying etiology remains incompletely understood. Among the mechanisms most strongly linked to hypertensive disorders of pregnancy, oxidative stress (OS) has emerged as a central contributor. Excessive production of reactive oxygen species (ROS) contributes to DNA damage, apoptosis, endothelial dysfunction, increased release of pro-inflammatory cytokines, and impaired anti-inflammatory responses. This narrative review summarizes current evidence regarding the role of OS in the pathophysiology of PE and explores the potential impact of antioxidant-based strategies for its prevention and management.

A comprehensive literature search was conducted in PubMed, Scopus, and Web of Science, published in English, up to December 2025. Priority was given to clinical studies involving women with PE, particularly those evaluating antioxidant supplementation or related therapeutic interventions.

Although it remains unclear whether OS represents a primary cause or secondary consequence of PE, accumulating evidence suggests that reduced antioxidant capacity and increased OS markers contribute to disease development and progression. Studies evaluating antioxidant-based interventions report biologically relevant effects; however, clinical outcomes remain heterogeneous. Overall, OS appears to play a key role in PE, highlighting the need for well-designed longitudinal and interventional studies to clarify causality and define the true therapeutic value of targeting redox pathways.

## INTRODUCTION

### Background

Oxidative stress (OS), defined as an imbalance between endogenous oxidant and antioxidant species^1^, is a fundamental factor in the development and progression of various pathologies, including hypertensive disorders¹. Hypertensive disorders of pregnancy encompass gestational hypertension, preeclampsia (PE), and eclampsia, and represent a leading cause of adverse maternal and perinatal outcomes worldwide^2,3^.

PE is a pregnancy-specific hypertensive disorder characterized by new-onset hypertension after 20 weeks of gestation, defined by systolic blood pressure ≥140 mmHg and/or diastolic blood pressure ≥90 mmHg, accompanied by proteinuria or, in its absence, evidence of maternal organ dysfunction^2,4^. Beyond this clinical definition, PE is increasingly recognized as a complex systemic condition involving maladaptive placental and maternal adaptive responses. Accumulating evidence suggests that alterations in placental development, immune regulation, angiogenic balance, and redox homeostasis contribute to its onset and clinical heterogeneity^3^. Within this multifactorial framework, disturbances in oxidative balance have been closely linked to endothelial dysfunction and adverse maternal–fetal outcomes^3^. These observations have stimulated growing interest in antioxidant-based approaches as potential preventive or therapeutic strategies in PE, although their clinical effectiveness remains under active investigation.

### Clinical diagnosis of preeclampsia

Hypertension and proteinuria constitute the classic diagnostic criteria for PE. However, additional clinical features are also considered, particularly in women with gestational hypertension in whom proteinuria is absent. In 2020, the American College of Obstetricians and Gynecologists (ACOG) updated the diagnostic and management criteria for hypertensive disorders of pregnancy^4^.

PE is primarily characterized by new-onset hypertension developing after 20 weeks of gestation^5^. Although the condition is frequently accompanied by proteinuria, defined as ≥300 mg of protein in a 24-hour urine collection, ≥2 on a dipstick test, or a protein-to-creatinine ratio >0.3, proteinuria may be absent in some cases (Table 1)^4^. In such instances, the diagnosis of PE is established based on the presence of hypertension together with evidence of maternal end-organ dysfunction, as defined by the severe features^2,4^ shown in Table 1.

**Table 1:** Severe features supporting the diagnosis of preeclampsia in the absence of proteinuria

Features	Indications
Thrombocytopenia	Platelet count <100.000×10^9^/L
Renal insufficiency	Serum creatinine >1.1 mg/dL or doubling of baseline creatinine in the absence of other renal disease
Impaired liver function	Hepatic transaminase levels were elevated to ≥2 times the upper limit of normal
Pulmonary involvement	Pulmonary edema not attributable to other causes
Neurological symptoms	New-onset headache unresponsive to medication or visual disturbances

### Epidemiology

PE and eclampsia constitute a major global health burden and remain the leading causes of maternal morbidity and mortality^2^. It is estimated that more than 50000 women die annually worldwide as a consequence of these hypertensive disorders of pregnancy^5^. PE affects 3–8% of all pregnancies, with a disproportionately higher prevalence and mortality observed in low- and middle-income countries, where it accounts for 9–26% of maternal deaths^2,4^.

Epidemiological evidence indicates that the incidence of PE is influenced by multiple factors, including ethnicity, family history (particularly a sororal history of PE), and population ancestry. Higher rates have been reported among African, American, and Hispanic women, whereas Asian populations appear to exhibit a comparatively lower risk. These disparities highlight the contribution of demographic, environmental, and genetic heterogeneity in the global distribution of the disease^6^.

### Etiology

The etiology of PE has not been fully elucidated; however, multiple factors have been implicated in its development. Genetic susceptibility plays a central role and includes polymorphisms in genes involved in blood pressure regulation, placental development, remodeling of uterine spiral arteries, immune response, and histocompatibility. These include *NPPA*, *NPPB*, *NPR3*, *PLCE1*, *TNS2*, *FURIN*, *RGL3*, *VEGF*, *HLA class I* and *KIR*^7-10^.

In addition to genetic factors, PE has been associated with a family history of hypertension, cardiovascular disease, renal disease, diabetes, obesity, unhealthy dietary patterns, advanced maternal age (aged ≥35 years), nulliparity, and multiple gestation^3,11^.

### Pathophysiological mechanisms of preeclampsia

During normal pregnancy, the progressive increase in fetal growth demands greater delivery of nutrients and oxygen, leading to enhanced placental blood perfusion^12^. To accommodate these uteroplacental hemodynamic changes, the maternal cardiovascular system undergoes physiological adaptations, including increased plasma volume and cardiac output^12,13^. In PE, however, these adaptive mechanisms are impaired, resulting in uteroplacental ischemia^12^.

This pathological condition is characterized by inadequate vascular remodeling, primarily due to shallow migration of cytotrophoblasts into the uterine spiral arteries. In contrast, normal pregnancy involves deep trophoblastic invasion, endothelial replacement, and differentiation into endothelioid cytotrophoblasts^14^. Defective vascular transformation ultimately leads to placental ischemia, inflammation, and apoptosis. Endothelial cells play a fundamental role in maintaining vascular homeostasis; therefore, endothelial dysfunction disrupts vasoprotective mechanisms and triggers a cascade of deleterious events, including the release of pro-inflammatory cytokines and antiangiogenic factors into the maternal circulation^15,16^.

One of the mechanisms proposed to explain endothelial dysfunction in PE involves antagonism of vascular endothelial growth factor (VEGF). VEGF is a critical regulator of angiogenesis and contributes to blood pressure reduction through the induction of vasodilatory mediators^17-19^. PE is increasingly recognized as an antiangiogenic state, characterized by elevated circulating levels of soluble fms-like tyrosine kinase 1 (sFlt-1) and endothelin 1, which contribute to systemic maternal vascular dysfunction^18^.

Accumulating evidence also indicates that overexpression of hypoxia-inducible transcriptional factor-1α (HIF-1α) in PE is associated with hypertension and proteinuria. HIF-1α overactivation promotes increased production of sFLT1, a potent antiangiogenic factor that antagonizes VEGF signaling and is consistently found at elevated levels in patients with PE^19^.

### Purpose of the review

This narrative review aims to synthesize current evidence regarding the role of OS in the pathophysiology of PE, with particular emphasis on its association with placental dysfunction, angiogenic imbalance, and endothelial alterations. In addition, it evaluates available experimental and clinical data on antioxidant-based strategies in PE, analyzing their potential benefits, limitations, and ongoing controversies. By integrating these findings, this review seeks to provide an updated perspective that enhances understanding of disease mechanisms and informs future research directions and potential therapeutic approaches.

A narrative literature review was conducted to summarize current evidence on OS and its role in the pathophysiology of PE, as well as to explore the potential effects of antioxidant-based strategies in its prevention and management. Relevant literature was identified through searches in PubMed, Scopus, and Web of Science using combinations of keywords and Medical Subjects Headings (MeSH) terms, including: ‘preeclampsia’, ‘oxidative stress’, ‘endothelial dysfunction’, ‘placental dysfunction’, ‘antioxidants’, and ‘antioxidant therapy’.

The search was limited to articles published up to December 2025 and restricted to studies in English. Priority was given to clinical studies involving women with PE, particularly those evaluating antioxidant supplementation or other therapeutic interventions. Studies addressing OS, endothelial dysfunction, and placental pathology in the context of PE were included to describe the available clinical evidence. Preclinical experimental studies were considered only when they provided relevant mechanistic insights supporting clinical observations. Case reports, editorials, conference abstracts lacking complete data, and publications not directly related to the scope of this review were excluded. In addition, the reference lists of all selected articles and relevant reviews were manually screened to identify additional pertinent publications.

## OXIDATIVE STRESS AND ANTIOXIDANTS IN PREECLAMPSIA

### Evidence supporting the role of oxidative stress in preeclampsia

The generation of reactive oxygen species (ROS) is a physiological consequence of aerobic metabolism, particularly mitochondrial oxidative phosphorylation, and is tightly regulated by endogenous antioxidant systems to maintain cellular homeostasis^20^. However, under pathological conditions such as hypoxia and ischemia, ROS production increases markedly, leading to redox imbalance and OS^21^. This imbalance contributes to damage of cellular macromolecules, including proteins, lipids, telomeric and genomic DNA, progressively impairing repair and defense mechanisms and favoring cellular senescence^21^.

In this context, ROS have been shown to induce the senescence-associated secretory phenotype (SASP), characterized by the release of pro-inflammatory cytokines, chemokines, growth factors, and proteases that influence tissue function and intercellular communication^21,22^. Increasing evidence suggests that senescence-related alterations play a relevant role in PE. Mesenchymal stem cells derived from women with PE display features of premature senescence and reduced angiogenic potential, whereas experimental targeting of senescence pathways has been reported to partially restore angiogenic function^21-25^. In line with these findings, Barbouti et al.^24^ have emphasized that OS-induced senescence may represent a critical component of placental dysfunction in PE, as sustained redox imbalance accelerates placental aging, enhances SASP signaling, and disrupts trophoblast function, thereby potentially contributing to disease progression.

Under physiological conditions, ROS are essential in biological processes such as immune regulation, cell proliferation and migration, and angiogenesis, which is required to ensure adequate oxygen supply at the placental level^15^. In PE, however, ROS production exceeds the capacity of antioxidant defense systems, and increased OS markers have consistently been observed in affected women^26^. Several studies have demonstrated that ROS-mediated damage to macromolecules, particularly lipid peroxidation, is significantly greater in women with both mild and severe PE compared with normotensive pregnant women, with more pronounced alterations observed in severe forms of the disease^26^.

Consistent with these observations, longitudinal clinical studies suggest that redox imbalance may precede the clinical onset of PE. Genc et al.^27^ reported that women who later developed PE exhibited higher levels of lipid peroxidation products and reduced antioxidant capacity during the first and second trimesters compared with normotensive pregnancies, indicating that OS alterations may contribute to early disease development rather than representing a late consequence.

Beyond its association with cellular senescence, oxidative stress (OS) in preeclampsia (PE) is closely linked to inflammatory activation and angiogenic dysregulation. The hypoxia–reperfusion cycles characteristic of PE promote excessive ROS generation through activation of xanthine oxidase and mitochondrial electron transport chain complexes I and III, contributing to vascular and placental dysfunction^13,28^. Oxidative stress has been shown to activate the nuclear factor-κB (NF-κB) pathway, a key mediator of inflammation that enhances the release of pro-inflammatory cytokines and antiangiogenic factors such as soluble fms-like tyrosine kinase-1 (sFlt-1)^3,13,18^. This pro-inflammatory milieu induces pro-apoptotic signaling and activates multiple pathogenic cascades characteristic of PE^21,24^.

Clinical studies have consistently reported increased circulating levels of inflammatory mediators, including tumor necrosis factor-α (TNFα) and interleukin (IL)-6, along with reduced concentrations of anti-inflammatory cytokines such as IL-10 and IL-4 in women with PE^3,14^. Notably, increased OS has been detected as early as 16–20 weeks of gestation in women who later develop PE, suggesting that excessive ROS production disrupts placental development and may contribute to disease programming^27,29^. More recent evidence further supports these findings, demonstrating elevated ROS and lipid peroxidation products, reduced antioxidant enzyme activity, and increased antiangiogenic factors in maternal blood and placental tissue from women with PE^30^.

**Figure 1:**
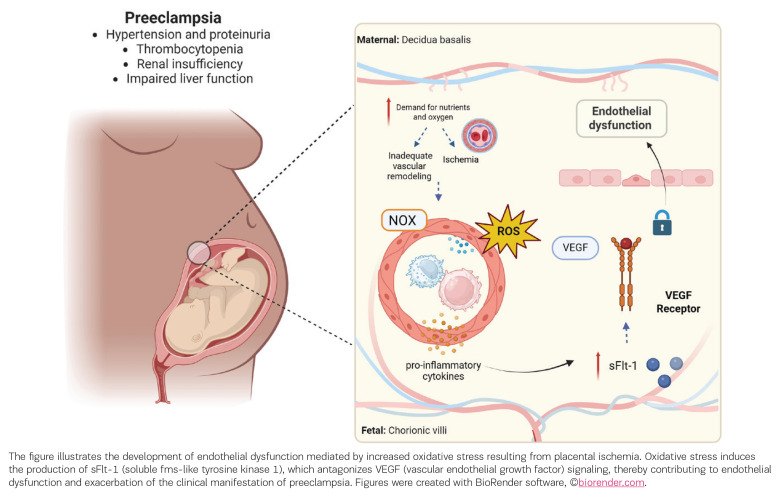
Proposed role of oxidative stress in the pathophysiology of preeclampsia, highlighting placental ischemia, endothelial dysfunction, inflammation, and dysregulation of angiogenic factors

Emerging data also suggest that OS may contribute to the progression of gestational hypertension to PE. In this context, some studies have proposed that the uric acid/superoxide dismutase (SOD) ratio is a potential biomarker for predicting PE development^31^. A systematic review and meta-analysis by Freire et al.^32^ evaluated OS markers across different PE subtypes, including early- and late-onset disease. The analysis found consistently higher lipid peroxidation levels and reduced antioxidant capacity in both maternal blood and placental tissue from preeclamptic pregnancies compared with normotensive controls^32^. Importantly, these alterations were observed across the disease spectrum, supporting the concept that redox imbalance is a fundamental pathogenic feature rather than a secondary consequence of clinical severity. The principal mechanisms linking OS to PE pathophysiology are summarized in Figure 1.

### Clinical evidence on antioxidant use in preeclampsia

The use of antioxidants as adjuvant therapy has shown favorable effects in conditions associated with increased OS, such as type 2 diabetes and metabolic diseases, including obesity, where supplementation has been linked to the attenuation or delayed progression of complications^33^. In PE, several studies have explored the potential benefits of antioxidant compounds, including selenium, an essential micronutrient with recognized antioxidant and immunomodulatory properties^34^.

Clinical trials have demonstrated that selenium supplementation for 12 weeks in high-risk pregnant women, initiated between 16 and 18 weeks of gestation, results in significantly increased serum selenium concentrations and improved overall antioxidant capacity compared with placebo^35^. In addition, reductions in high-sensitivity C-reactive protein levels, improvements in metabolic parameters, and favorable effects on uterine artery pulsatility index have been reported, suggesting a potential protective role of selenium in women at increased risk of developing PE^35^.

Another antioxidant that has been studied in the context of PE is resveratrol, a plant-derived polyphenolic stilbene widely recognized for its antioxidant, anti-inflammatory, and vasoprotective effects, commonly found in grapes, berries, and red wine^36^. Clinical evidence indicates that resveratrol supplementation in combination with standard antihypertensive therapy, such as nifedipine, significantly improves blood pressure control^37^. Randomized controlled trials have shown that the nifedipine–resveratrol combination shortens the time required to achieve target blood pressure, delays recurrent hypertensive episodes, and reduces the total number of antihypertensive doses needed compared with nifedipine plus placebo, without significant maternal or neonatal adverse effects^37^. These findings suggest a potential therapeutic benefit associated with the antioxidant and endothelial-protective effects of resveratrol. Additional studies in overweight pregnant women have reported reduced incidence of gestational diabetes, improved lipid and glycemic profiles, and improved blood pressure control in women with PE receiving resveratrol supplementation^38^.

Other antioxidant-related interventions have yielded mixed results. Oral magnesium citrate supplementation (300 mg/day) did not reduce the incidence of PE in a controlled clinical trial involving 159 pregnant women per study group^39^. Similarly, a large randomized, double-blind, multicenter trial evaluating high-dose folic acid supplementation (4.0 mg/day) from early pregnancy until delivery in high-risk women found no significant reduction in PE incidence compared with placebo^40^.

In contrast, coenzyme Q10 (CoQ10), a key component of mitochondrial electron transport and antioxidant defense, has shown promising results. In a randomized controlled clinical trial, supplementation with 200 mg/day of CoQ10 from 20 weeks of gestation until delivery significantly reduced the incidence of PE in high-risk pregnant women^41^. Supporting these findings, reduced maternal and placental CoQ10 levels have been reported in women with severe PE, further implicating mitochondrial dysfunction and OS in disease severity^42^.

Given the central role of OS and inflammatory dysregulation in PE, additional nutritional strategies have also been explored. In a study evaluating calcium supplementation over six weeks in high-risk pregnant women, improvements in antioxidant activity, modulation of the purinergic system, and reductions in inflammatory cytokines were observed, suggesting a potential adjunctive benefit of calcium in modulating OS-related pathways^43^.

OS is widely recognized as a central component in the pathophysiology of PE, as disturbances in redox homeostasis appear to contribute both to placental dysfunction and systemic endothelial impairment. Previous reviews have emphasized that excessive production of ROS promotes placental ischemia, trophoblastic injury, inflammatory activation and angiogenic imbalance, thereby positioning OS as a key biological axis in placental disorders^44,45^. Although current evidence strongly supports its dynamic involvement in disease development, it remains uncertain whether OS acts primarily as an initiating factor, an amplifying mechanism, or a downstream consequence of placental dysfunction^27^.

Nevertheless, accumulating data increasingly suggest that redox alterations may arise early in pregnancy, potentially preceding overt clinical manifestations of PE. This association highlights the need for robust longitudinal studies and standardized oxidative stress biomarkers to clarify causality, define the timing of redox alterations, and determine the true preventive or therapeutic relevance of targeting oxidative pathways. In this regard, OS has also been implicated in disease progression, particularly in the transition from gestational hypertension to PE. For example, the uric acid/SOD ratio has been proposed as a predictive indicator of PE development^31^, reinforcing the hypothesis that disruption in redox balance may precede clinical deterioration.

Further supporting this concept, a recent systematic review and meta-analysis by Freire et al.^32^ demonstrated consistently elevated lipid peroxidation markers together with reduced antioxidant capacity in both maternal blood and placental tissue from women with PE compared with normotensive controls. Importantly, these alterations were observed across different PE phenotypes, including early-onset and severe forms, suggesting that oxidative imbalance is a shared and sustained feature across the disease spectrum rather than a secondary late event^32^.

Within this context, the growing understanding of OS as a key contributor to PE pathophysiology has stimulated interest in antioxidant-based interventions as potential preventive or therapeutic strategies. Available clinical evidence suggests that correcting redox imbalance may exert beneficial systemic effects. For instance, selenium supplementation (an essential micronutrient involved in redox regulation and protection against oxidative damage) has been associated with significant reductions in high-sensitivity C-reactive protein levels, along with improvements in metabolic parameters and uterine artery pulsatility index in high-risk pregnant women. Similarly, resveratrol has emerged as a promising antioxidant compound with vascular and endothelial-protective properties, showing favorable effects on blood pressure control and inflammatory markers in selected clinical settings.

However, antioxidant interventions have not produced uniformly positive results. For example, magnesium citrate supplementation failed to reduce the incidence of PE in a randomized clinical trial, underscoring that antioxidant therapy is not universally effective. These heterogeneous findings suggest that the clinical impact of antioxidant strategies likely depends on multiple factors, including the specific compound used, its biological target, timing of administration, doses, and the underlying risk profile of the patient population. Further research with a systematic review approach is needed to further elucidate the association between OS and preeclampsia.

## CONCLUSION

Clinical evidence regarding antioxidant supplementation in pregnant women with PE remains inconsistent. While some antioxidants, such as selenium, resveratrol, and CoQ10, have shown potential benefits by reducing the incidence or severity of PE, other interventions have failed to demonstrate consistent clinical efficacy. Although folic acid may indirectly modulate redox balance by increasing glutathione (GSH) levels, enhancing total antioxidant capacity (TAC), and reducing malondialdehyde (MDA), randomized clinical trials have shown that high-dose folic acid supplementation does not reduce the incidence of PE. Similarly, vitamin C supplementation has yielded conflicting results and, in some cases, has been associated with adverse outcomes, including an increased risk of intrauterine growth restriction.

This variability in clinical response may be explained by differences in the molecular mechanisms, biological targets, and pharmacological properties of each antioxidant, as well as their capacity to modulate endothelial dysfunction and OS-related pathways. Therefore, further well-designed and adequately powered clinical studies are needed to clarify the role of antioxidant-based interventions and to identify those strategies most likely to provide meaningful preventive or therapeutic benefits in PE.

## Data Availability

Data sharing is not applicable to this article as no new data were created.
